# Hinokitiol Exhibits Antitumor Properties through Induction of ROS-Mediated Apoptosis and p53-Driven Cell-Cycle Arrest in Endometrial Cancer Cell Lines (Ishikawa, HEC-1A, KLE)

**DOI:** 10.3390/ijms22158268

**Published:** 2021-07-31

**Authors:** Hsin-Yuan Chen, Wen-Pin Cheng, Yi-Fen Chiang, Yong-Han Hong, Mohamed Ali, Tsui-Chin Huang, Kai-Lee Wang, Tzong-Ming Shieh, Hsin-Yi Chang, Shih-Min Hsia

**Affiliations:** 1 School of Nutrition and Health Sciences, College of Nutrition, Taipei Medical University, Taipei 11031, Taiwan; hsin246@gmail.com (H.-Y.C.); yvonne840828@gmail.com (Y.-F.C.); 2 Department of Nutrition, I-Shou University, Kaohsiung 84001, Taiwan; yonghan@isu.edu.tw; 3 Department of Medical Education and Research, Shin Kong Wu Ho-Su Memorial Hospital, Taipei 11101, Taiwan; T011788@ms.skh.org.tw; 4 Clinical Pharmacy Department, Faculty of Pharmacy, Ain Shams University, Cairo 11566, Egypt; mohamed.aboouf@pharma.asu.edu.eg; 5 Graduate Institute of Cancer Biology and Drug Discovery, College of Medical Science and Technology, Taipei Medical University, Taipei 11031, Taiwan; tsuichin@gmail.com; 6 Department of Nursing, Ching Kuo Institute of Management and Health, Keelung 20301, Taiwan; kellywang@tmu.edu.tw; 7 School of Dentistry, College of Dentistry, China Medical University, Taichung 40402, Taiwan; tmshieh@mail.cmu.edu.tw; 8 Graduate Institute of Metabolism and Obesity Sciences, College of Nutrition, Taipei Medical University, Taipei 11031, Taiwan; hsinyi.chang@tmu.edu.tw; 9 School of Food and Safety, Taipei Medical University, Taipei 11031, Taiwan; 10 Nutrition Research Center, Taipei Medical University Hospital, Taipei 11031, Taiwan

**Keywords:** hinokitiol, endometrial cancer, apoptosis, reactive oxygen species

## Abstract

Hinokitiol is a natural tropolone derivative that is present in the heartwood of cupressaceous plants, and has been extensively investigated for its anti-inflammatory, antioxidant, and antitumor properties in the context of various diseases. To date, the effects of hinokitiol on endometrial cancer (EC) has not been explored. The purpose of our study was to investigate the anti-proliferative effects of hinokitiol on EC cells. Cell viability was determined with an MTT (3-(4,5-Dimethylthiazol-2-yl)-2,5-diphenyltetrazolium bromide) assay, and the quantification of apoptosis and reactive oxygen species (ROSs) was performed by using flow cytometry, while protein expression was measured with the Western blotting technique. Hinokitiol significantly suppressed cell proliferation through the inhibition of the expression of cell-cycle mediators, such as cyclin D1 and cyclin-dependent kinase 4 (CDK4), as well as the induction of the tumor suppressor protein p53. In addition, hinokitiol increased the number of apoptotic cells and increased the protein expression of cleaved-poly-ADP-ribose polymerase (PARP) and active cleaved-caspase-3, as well as the ratio of Bcl-2-associated X protein (Bax) to B-cell lymphoma 2 (Bcl-2). Interestingly, except for KLE cells, hinokitiol induced autophagy by promoting the accumulation of the microtubule-associated protein light chain 3B (LC3B) and reducing the sequestosome-1 (p62/SQSTM1) protein level. Furthermore, hinokitiol triggered ROS production and upregulated the phosphorylation of extracellular-signal-regulated kinase (p-ERK1/2) in EC cells. These results demonstrate that hinokitiol has potential anti-proliferative and pro-apoptotic benefits in the treatment of endometrial cancer cell lines (Ishikawa, HEC-1A, and KLE).

## 1. Introduction

The prevalence of endometrial cancer (EC) has rapidly risen worldwide in recent years, including in the United States [1] and in Taiwan [2]. It is estimated that 417,367 new cases of and 97,370 deaths from corpus uteri (also known as endometrial cancer) happened worldwide in 2020 [3]. In general, EC has been histologically classified as either type 1 EC (well-differentiated) or type 2 EC (poorly differentiated), depending on the presence of estrogen. In addition, according to its different clinical features, EC can be subdivided into (i) endometrioid endometrial cancer (EEC, or type 1), (ii) serous endometrial cancer (SEC, or type 2), (iii) clear-cell endometrial cancer (CCEC, or type 2), and (iv) mixed endometrial cancer and uterine carcinoma (USC) [4,5]. To strengthen observers’ consensus on the histological classification of tumors and to improve the prognosis of EC patients, many studies have been dedicated to incorporating molecular classification with The Cancer Genome Atlas (TCGA) analysis, which includes TP53 mutations, into routine clinical histological diagnostic evaluations [6]. Mutations in p53 lead to the loss of tumor suppressor activity while promoting the survival and proliferation of malignant tumors [7].

**Figure 1:**
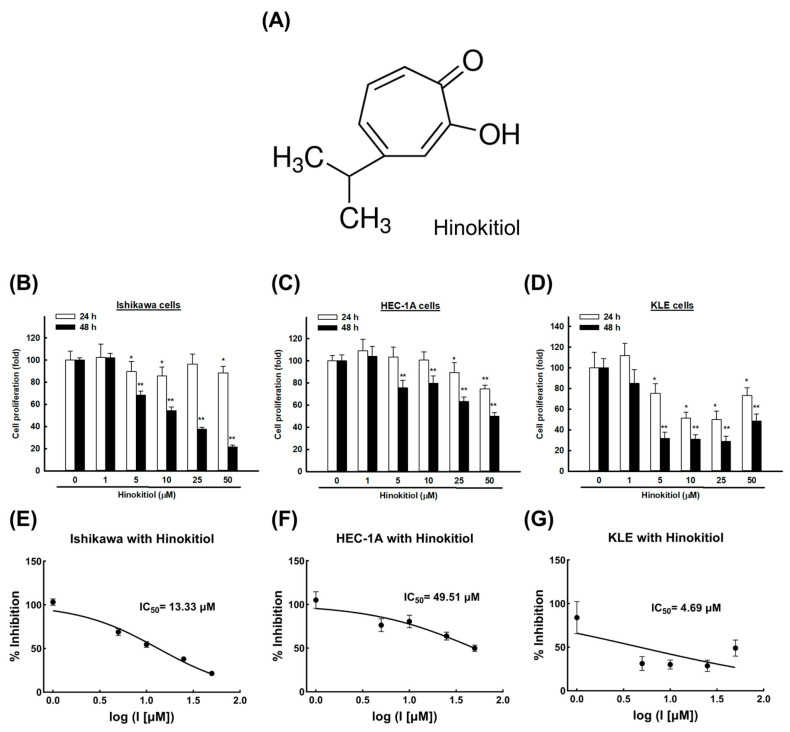
The growth-inhibitory effect of hinokitiol on endometrial cancer cells. (**A**) Chemical structure of hinokitiol. Ishikawa (**B**), HEC-1A (**C**), and KLE (**D**) cells were treated with hinokitiol (0, 1, 5, 10, 25, and 50 µM) for 24 or 48 h; cell viability was evaluated with an MTT assay. (**E**–**G**) Dose–response curve and half-maximal inhibitory concentration (IC_50_) values of hinokitiol in endometrial cancer cells. Data are expressed as the mean ± SD of three independent experiments; * *p* < 0.05 and ** *p* < 0.001 compared to the control group.

Considering the side effects caused by chemotherapy and radiotherapy, it is critical to seek alternative treatments for malignant cancers, including endometrial cancer. Naturally occurring compounds have a variety of effective phytochemicals and usually have lower toxicity than chemotherapy, so they are widely used in the clinical treatment of various cancers [8,9]. Hinokitiol, also known as β-Thujaplicin, is a natural tropolone derivative with a seven-membered carbon ring and an isopropyl side chain (Figure 1A). It is widely used in cosmetics and oral-care products due to its strong antifungal and antimicrobial activities.

A large amount of the literature in the public domain has demonstrated the various biological properties of hinokitiol, including its neuroprotective activity [10], as well as its antibacterial [11] and anti-inflammatory [12] effects. In addition, hinokitiol has been shown to have antitumor potential against a variety of cancers; this potential involves reducing metastasis in 4T1 breast cancer cells [13], promoting apoptosis in A549 lung cancer cells [14], inducing senescence and autophagy in HeLa cervical cancer cells [15], disrupting androgen receptor signaling in LNCaP prostate carcinoma cells [16], and alleviating the migration ability [13] and melanogenesis in B16F10 melanoma cells [17]. It is worth mentioning that hinokitiol has the ability to inhibit tumor growth without causing damage to normal tissue; for example, hinokitiol simultaneously inhibits the growth of oral pathogens and oral squamous cell carcinoma cells without inducing toxicity in normal human oral keratinocytes [18].

Although many studies have been conducted on the beneficial effects of hinokitiol on cancer, its antitumor activity and the exact pathophysiological mechanism with respect to EC have not been investigated. Therefore, in this study, we focus on the inhibitory effects of hinokitiol on EC through its effects on cell proliferation, apoptosis, and autophagy.

## 2. Results

### 2.1. Hinokitiol Induces an Anti-Proliferative Effect on Endometrial Cancer Cells

To evaluate the cytotoxicity of hinokitiol, we treated human endometrial cancer cell lines (Ishikawa, HEC-1A, and KLE) with different concentrations of hinokitiol (1, 5, 10, 25, and 50 μM) or dimethyl sulfoxide (DMSO) for 24 and 48 h. Then, 3-(4,5-dimethylthiazol-2-yl) -2,5-diphenyltetrazolium bromide (MTT) assays were used to analyze the cell viability. As shown in Figure 1B–D, starting from the dose of 5 μM, hinokitiol induced a sharp decline in the cell viability and significantly inhibited the growth of the Ishikawa, HEC-1A, and KLE cells compared with the DMSO control, especially after 48 h (*p* < 0.001). The IC_50_ after treatment for 48 h was estimated to be 13.33 μM in the Ishikawa cells, 49.51 μM in the HEC-1A cells, and 4.69 μM in the KLE cells (Figure 1E–G).

### 2.2. Hinokitiol Promotes Cell-Cycle Arrest at the G0/G1 Phase in Endometrial Cancer Cells

**Figure 2:**
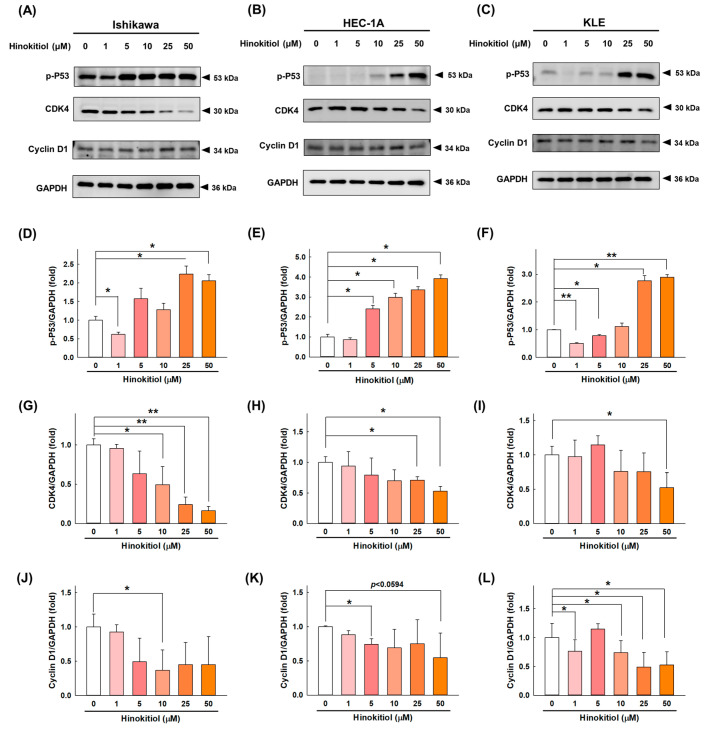
Hinokitiol’s effect on cell-cycle-related proteins in endometrial cancer cells. Ishikawa (**A**), HEC-1A (**B**), and KLE (**C**) cells were treated with hinokitiol (0, 1, 5, 10, 25, and 50 µM) for 48 h, and the protein expression of p-P53 (**D**–**F**), CDK4 (**G**–**I**), and cyclin D1 (**J**–**L**) was assayed through Western blotting analysis. The differences in target proteins were analyzed with Sigma Plot, and data are expressed as the mean ± SD of three independent experiments; * *p* < 0.05 and ** *p* < 0.001 compared to the control group.

To assess whether hinokitiol has an effect on the progression of the EC cell cycle, we examined the expression of regulatory proteins of the cell cycle after hinokitiol treatment. Our data revealed that hinokitiol doses of greater than 25 μM significantly upregulated the cell-cycle inhibitor p53 in the Ishikawa (Figure 2A,D), HEC-1A (Figure 2B,E), and KLE (Figure 2C,F) cells. In addition, hinokitiol significantly inhibited the expression of the cell-cycle regulatory proteins CDK4 and cyclin D1 in the Ishikawa (Figure 2G,J), HEC-1A (Figure 2H,K), and KLE (Figure 2I,L) cells. Consequently, we propose that hinokitiol induced an increase in the p53 level and a decrease in cyclin D1 and CDK4 expression, which implies that hinokitiol affected the growth of EC cells through the induction of the arrest of the G0/G1 phase.

### 2.3. Hinokitiol Induces Cell Apoptosis via the Caspase Apoptotic Pathway in Endometrial Cancer Cells

**Figure 3:**
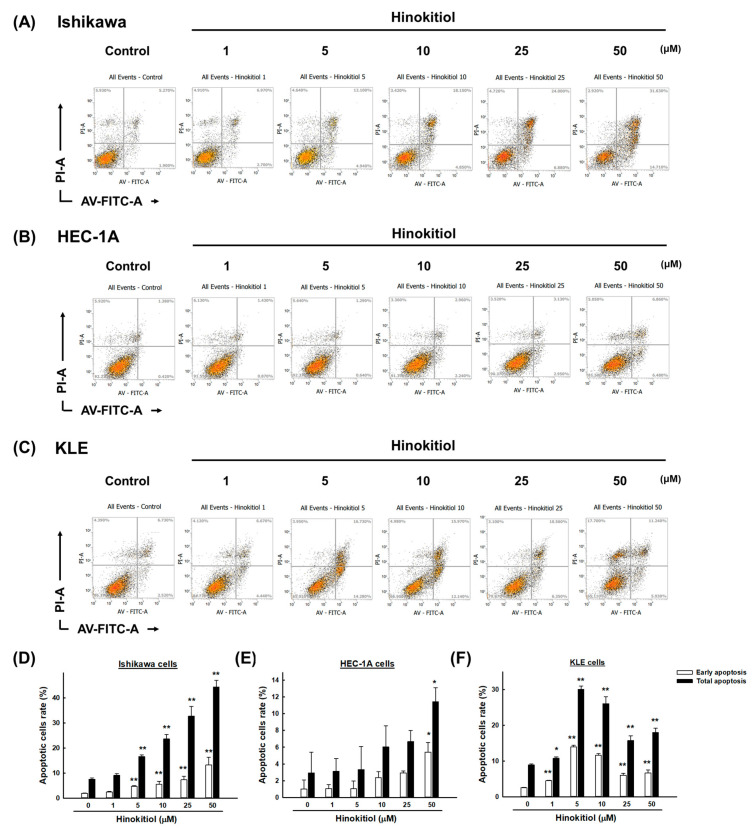
Hinokitiol’s effect on cell apoptosis in endometrial cancer cells. Ishikawa (**A**), HEC-1A (**B**), and KLE (**C**) cells were treated with hinokitiol (0, 1, 5, 10, 25, and 50 µM) for 48 h, and cell death was detected by using Annexin V-FITC/PI double staining with flow cytometry. The differences in the distribution of early and total apoptotic cells were analyzed with a Sigma Plot (**D**–**F**), and the data are expressed as the mean ± SD of three independent experiments; * *p* < 0.05, ** *p* < 0.001 compared to the control group. For (**A**–**C**), left lower left: healthy cells; lower right: early apoptosis; upper right: late apoptosis; upper left: necrotic cells.

Accumulating evidence has suggested that the activation of the p53/p21 pathway promotes apoptosis, and this pathway is part of programmed cell death [19]. To explore whether hinokitiol triggers cell apoptosis, the percentages of apoptotic cells after the treatment were examined by using Annexin-V/PI staining. As the results showed, the numbers of cells in the early apoptosis (annexin V+/PI−) and late apoptosis (annexin V+/PI+) phases increased after the hinokitiol treatment. Interestingly, the treatment had different effects depending on the type of cell line. In detail, in the HEC-1A cells, only the dose of 50 μM significantly increased the number of apoptotic cells, while lower doses showed statistically insignificant increases (Figure 3B,E); However, in the Ishikawa cells, doses of hinokitiol greater than 5 μM significantly increased the number of apoptotic cells (Figure 3A,D). Finally, in the KLE cells, significant induction of apoptosis appeared starting from the dose of 1 μM hinokitiol; the dose of 5 μM achieved the greatest pro-apoptotic effect (Figure 3C,F).

**Figure 4:**
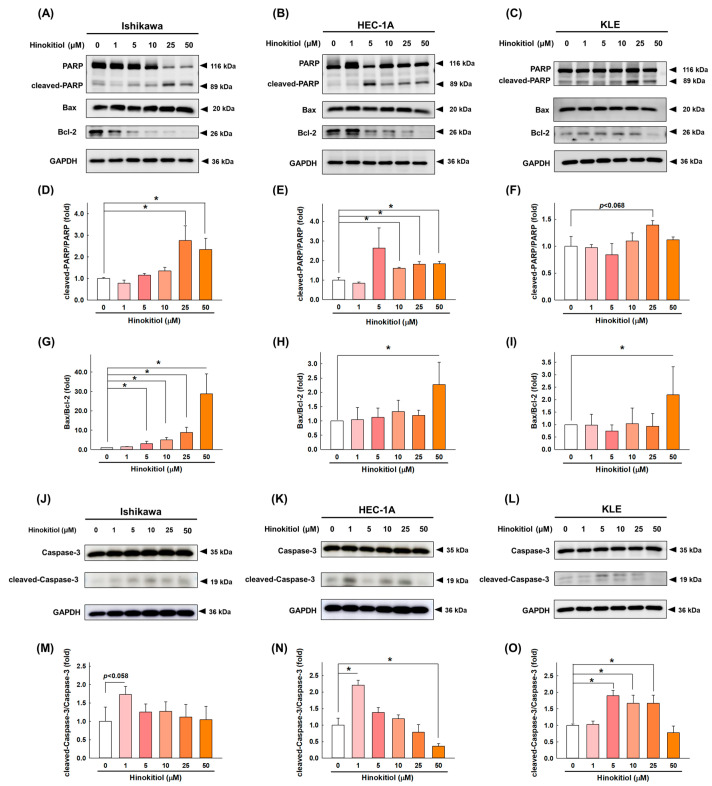
Hinokitiol’s effect on cell-apoptosis-related protein expression in endometrial cancer cells. Ishikawa (**A**,**J**), HEC-1A (**B**,**K**), and KLE (**C**,**L**) cells were treated with hinokitiol (0, 1, 5, 10, 25, and 50 µM) for 48 h, and the protein expression of PARP (**D**–**F**), Bax, Bcl-2 (**G**–**I**), and caspase-3 (**M**–**O**) was assayed with a Western blotting analysis. The differences in the target proteins were analyzed with a Sigma Plot, and the data are expressed as the mean ± SD of three independent experiments; * *p* < 0.05 compared to the control group.

To investigate the molecular mechanisms of hinokitiol-mediated apoptosis in endometrial cancer cells, the expression of the activated PARP, Bax, Bcl-2, and caspase-3 proteins was analyzed with a Western blotting assay. As shown in Figure 4A–C, regardless of the cell line, hinokitiol significantly increased the expression of cleaved PARP (Figure 4D–F), and the ratio of the pro-apoptotic protein Bax and anti-apoptotic protein Bcl-2 was also significantly increased after the hinokitiol treatment (Figure 4G–I). In addition, the expression of cleaved caspase-3 after hinokitiol treatment has different increasing trends in different endometrial cell lines. Although it did not increase with increasing doses, the expression of cleaved caspase-3 in Ishikawa (Figure 4J,M), HEC-1A (Figure 4K,N), and KLE (Figure 4L,O) cells increased significantly at specific doses. Collectively, these data suggest that hinokitiol might induce EC cell death by altering the activation of caspase.

### 2.4. Hinokitiol Induces Cell Autophagy in Endometrial Cancer Cells

**Figure 5:**
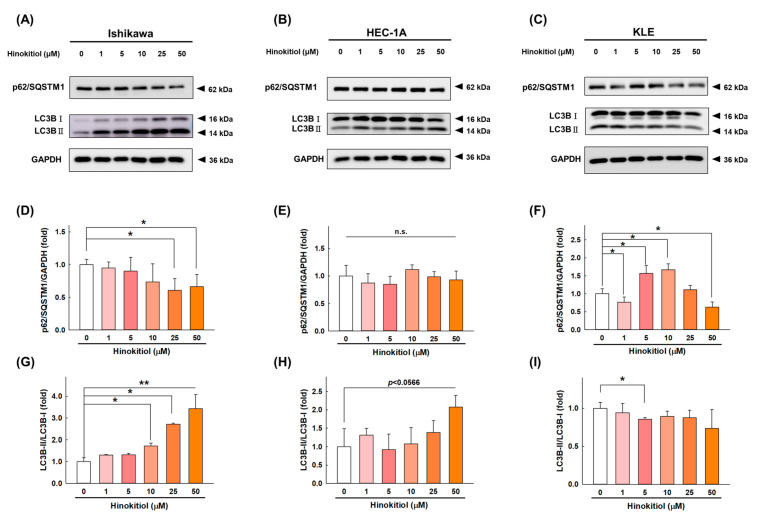
Hinokitiol’s effect on autophagy-related proteins in endometrial cancer cells. Ishikawa (**A**), HEC-1A (**B**), and KLE (**C**) cells were treated with hinokitiol (0, 1, 5, 10, 25, and 50 µM) for 48 h, and the protein expression of p62/SQSTM1 (**D**–**F**) and LC3BII (**G**–**I**) was assayed with a Western blotting analysis. Data are expressed as the mean ± SD of three independent experiments; n.s.: not significant; * *p* < 0.05 and ** *p* < 0.001 compared to the control group.

Programmed cell death also occurs through autophagy, which is a complex catabolic process that degrades damaged organelles and proteins through lysosome-mediated degradation and promotes nutrient circulation [20]. We next evaluated whether hinokitiol induces autophagy in EC cells. We assessed the levels of two autophagy regulators, p62/SQSTM1 and LC3, after the treatment with hinokitiol. As shown in Figure 5, after the intervention of hinokitiol, LC3-I was converted into LC3-II, causing the accumulation of LC3-II expression in the Ishikawa (Figure 5A,G), HEC-1A (Figure 5B,H) cells. However, no increasing trend was seen in KLE cells (Figure 5C,I). As the degradation substrate of autophagic membranes, the expression of p62/SQSTM1 was decreased after the treatment with hinokitiol in the Ishikawa (Figure 5A,D) and KLE (Figure 5C,F) cells. However, only a decreasing trend can be observed in HEC-1A cells (Figure 5B,E), but there is no statistical difference. Collectively, the accumulation of LC3-II and the downregulation of the p62 expression level after the hinokitiol treatment might be interpreted as the induction of autophagy.

### 2.5. Hinokitiol Induces Intracellular Reactive Oxygen Species (ROSs) in Endometrial Cancer Cells

**Figure 6:**
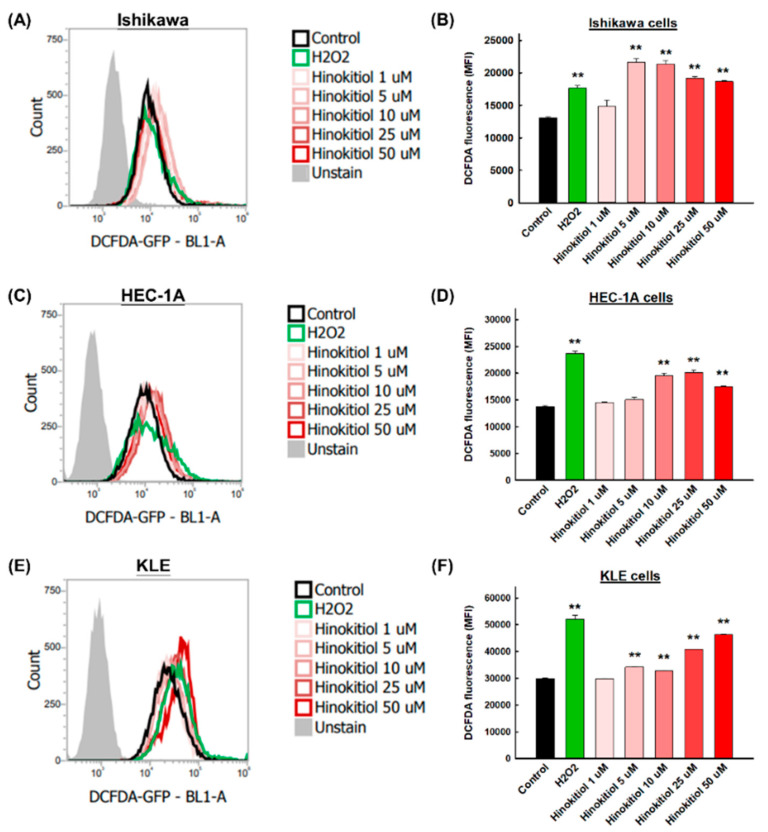
Hinokitiol’s effect on intracellular ROSs in endometrial cancer cells. Ishikawa (**A**), HEC-1A (**C**), and KLE (**E**) cells were treated with hinokitiol (0, 1, 5, 10, 25, and 50 µM) for 48 h, and the intensity of fluorescence was assayed with H_2_DCFDA staining (**B**,**D**,**F**). Data are expressed as the mean ± SD of three independent experiments; ** *p* < 0.001 compared to the control group.

To verify that natural products can activate reactive oxygen species (ROSs) and subsequently induce apoptosis in cancer cells, staining of the oxidative stress indicator H_2_DCFDA was used. H_2_O_2_ was used as the positive control group to confirm the induction of intracellular ROSs. Figure 6 shows representative profiles of ROS generation in the Ishikawa (Figure 6A), HEC-1A (Figure 6C), and KLE cells (Figure 6E). As in the positive control group, hinokitiol had the ability to enhance the fluorescence intensity in the three types of cells. Starting from 5 μM, hinokitiol significantly increased the production of ROSs in the Ishikawa cells (Figure 6B) and KLE cells (Figure 6F); starting from 10 μM, it significantly increased ROS production in the HEC-1A cells (Figure 6D).

### 2.6. Hinokitiol Regulates the ERK Signaling Pathway

**Figure 7:**
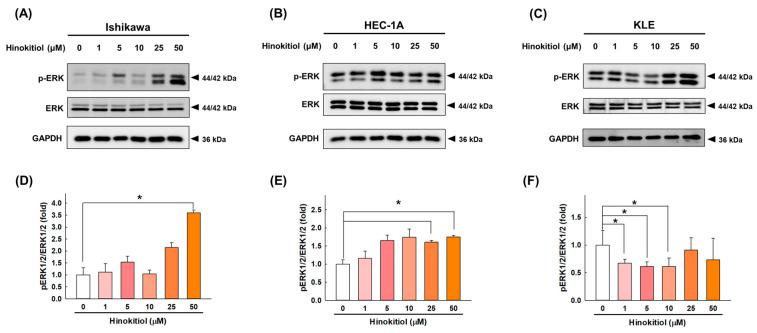
Hinokitiol’s effect on the activation of the ERK1/2 pathway in endometrial cancer cells. Ishikawa (**A**), HEC-1A (**B**), and KLE (**C**) cells were treated with hinokitiol (0, 1, 5, 10, 25, and 50 µM) for 48 h, and the expression of p-ERK1/2 was assayed through Western blotting, normalized with an internal control, and quantified with the total ERK1/2 (**D**–**F**). Data are expressed as the mean ± SD of three independent experiments; * *p* < 0.05 compared to the control group.

To determine whether the MAPK pathway is involved in the induction of apoptosis as one of hinokitiol’s effects, we measured the changes in ERK1/2 in response to treatment in endometrial cancer cells. As shown in Figure 7, hinokitiol significantly increased the phosphorylation of ERK1/2 at a dose of 50 μM in the Ishikawa cells (Figure 7A,D) and at doses of 25 and 50 μM in the HEC-1A cells (Figure 7B,E), while it significantly decreased the phosphorylation of ERK1/2 at doses of 1, 5, and 10 μM in the KLE cells (Figure 7C,F) in comparison with the untreated control. These results indicate that hinokitiol could trigger the activation of the ERK pathway in Ishikawa and HEC-1A cells, while it affected KLE cells through another signaling pathway.

## 3. Discussion

Accumulating evidence has suggested that the selective induction of cancer cell apoptosis is probably one of the most powerful cancer prevention strategies [9]. Our study revealed that hinokitiol possesses anti-cancer effects with respect to endometrial cancer cells, including Ishikawa, HEC-1A, and KLE cells, via the induction of tumor suppressor protein p53, apoptotic markers (Bax, PARP, and caspase-3), autophagic markers (LC3BII), and ROS levels, while it downregulates cell-cycle-related proteins (cyclin D1 and CDK4 and anti-apoptotic protein Bcl-2 and possibly regulates the ERK signaling pathway.

Cell proliferation is achieved through repeated cellular divisions and entry into the cell cycle, where each G0/G, S, and G2/M checkpoint is strictly controlled by cyclin and cyclin-dependent kinases (CDKs) [21]. If the cell cycle of cancer cells loses control of the checkpoints, their ability to proliferate will be affected, thus inhibiting cancer cell proliferation [22]. Wang et al. demonstrated that hinokitiol induced cell-cycle arrest at the G1 phase and significantly downregulated the protein levels of cyclin D1 and cyclin E in a dose-dependent manner in cervical carcinoma HeLa cells [15]. In addition, Chen et al. revealed that β-thujaplicin induced G0/G1 cell-cycle arrest and significantly inhibited cyclin D1, cyclin E, and CDK4 expression in a dose-dependent manner in MCF10DCIS.com breast cancer cells [23]. Our study showed similar results, where hinokitiol treatment for 48 h significantly reduced the expression of cyclin D1 and CDK4 in endometrial cancer cells.

The tumor suppressor p53 is a redox-active transcription factor that maintains genome stability and integrity by regulating diverse cellular functions, including cell-cycle arrest, DNA repair, senescence, and apoptosis [24]. The increase in the p53 level not only inhibits the expression of cyclin during the transition from the G1 to the S phase, but also causes an interaction with anti-apoptotic proteins (Bcl-2 homologous antagonist/killer, Bak; Bcl-2-associated X protein, Bax) that are located in the mitochondria to promote cell apoptosis [24]. We explored the expression of the p53 protein in response to treatment by using Western blotting, and our study showed that hinokitiol prominently induced the expression of p53 phosphorylation in Ishikawa, HEC-1A, and KLE cells. The results of previous reports are consistent with this observation, as hinokitiol increased the p53 protein level in A549 human lung cancer cells [14]. In addition, an increase in both the p21 and p53 levels was observed in hinokitiol-treated cervical carcinoma HeLa cells [15].

Apoptosis is characterized by cell death in the body through both external and internal pathways; the latter is also known as the mitochondria-dependent pathway [25]. Briefly, overproduction of ROSs downregulates B-cell lymphoma 2 (Bcl-2), while upregulation of Bcl-2-associated X protein (Bax) leads to increased mitochondrial outer membrane permeabilization (MOMP; loss of ΔΨm), triggers the release of mitochondrial cytochrome C into the cytoplasm, and activates a series of caspases, which, in turn, promote apoptosis [25]. Zhang et al. showed that β-Thujaplicin triggered HepG2 apoptosis and increased cleaved PARP1, cleaved caspase-3, and the Bax/Bcl-2 ratio, which indicated that β-Thujaplicin-induced apoptosis is mediated by the mitochondria-dependent pathway [26]. Similarly, our study found that the total number of apoptotic cells largely increased upon the administration of hinokitiol, and simultaneously, the expression of cleaved-PARP, cleaved-caspase-3, and the Bax/Bcl-2 ratio also increased in Ishikawa, HEC-1A, and KLE cells at 48 h, suggesting that the apoptosis induced by hinokitiol was possibly mediated by the mitochondrion-dependent pathway. It’s worth mentioning that over-degraded protein may be the reason why the expression of caspase-3 cannot be enhanced with increasing dose, or it may be a non-dominant caspase protein, which requires more experimental verification.

Autophagy is a pivotal step in tissue damage that is regulated in a manner of homeostatic balance between the degradation of damaged organelles and protein aggregates, and the recycling of nutrients [20]. The key biomarkers for autophagic flux are the microtubule-associated protein light chain 3 (LC3) and p62 (sequestosome-1, SQSTM1). During autophagy, LC3-II, which is converted from LC3-I, is recruited and incorporated into the autophagosome membrane. p62/SQSTM1 acts as a degradation substrate with respect to the autophagic membrane, usually interacts with LC3-II and polyubiquitin, and drives the occurrence of autophagy [27]. Hinokitiol has been shown to promote autophagy in different tissues. A previous study reported that β-Thujaplicin promoted the expression of LC3B-II and decreased the p62 protein level in HepG2 cells, indicating that autophagic events were increased upon β-Thujaplicin treatment [26]. Similarly, our study found that hinokitiol reduced the expression of p62/SQSTM1 and increased the LC3BII/I ratio in Ishikawa, HEC-1A, and KLE cells, indicating that hinokitiol possesses the ability to promote autophagy in endometrial cancer cells. It’s worth mentioning that the high expression of LC3B-II protein in the control group suggests that the KLE cells may have been in an autophagy state and cannot be induced anymore.

Excessive ROS can cause damage to cells through mechanisms that involve cell apoptosis, autophagy, and cell arrest [28]. In normal cells, hinokitiol was able to inhibit the H_2_O_2_-induced generation of ROSs, as shown in a study on human corneal epithelial cells, where 100 μmol of hinokitiol significantly suppressed ROS production in human corneal epithelium cells [29]. However, there are distinct results in cancer cells. Recent studies reported that ROS generation is increased in HepG2 cells after exposure to β-Thujaplicin [26]. Several studies have focused on the ROS-mediated apoptosis pathway and explored the effects of natural compounds on the growth of endometrial cancer [30,31]. In the current study, we used H_2_O_2_ as a positive control and found that hinokitiol could promote the production of ROSs in the three endometrial cancer cell lines used, and the effect was even greater than that of the positive control group. Thus, we speculate that hinokitiol may inhibit the growth of endometrial cancer through ROS-mediated apoptosis.

The mechanism of action of hinokitiol involves a variety of signaling pathways—for example, hinokitiol activates the ERK/MKP-3/proteosome pathway to inhibit the growth of B16F10 melanoma [17]. In addition, hinokitiol activates the AKT/GSK-3β/β-catenin signaling cascade to inhibit the growth of ER-negative MCF10DCIS.com human breast cancer cells [23]. As originally known, the ERK signaling pathway plays a pivotal role in cell survival. A review revealed that DNA damage can promote ERK activation and leads to p53-mediated cellular responses [32], which can even lead to an intrinsic apoptotic pathway, resulting in cell death. However, it is still controversial whether ERK expression is positively or negatively regulated after treatment with natural compounds. Interestingly, in the current study, we observed a phenomenon in which hinokitiol enhanced the expression of phosphorylated ERK1/2 in Ishikawa and HEC-1A cells while reducing it in KLE cells. The results of previous reports are consistent with the observation of increased ERK1/2 phosphorylation in β-Thujaplicin-treated HepG2 cells [26], which suggests that β-Thujaplicin can induce apoptosis via the ERK1/2 pathway.

The advantage of this experiment is to use different types of endometrial cancer cell lines to verify the effect of hinokitiol. In terms of histological classification, Ishikawa belongs to type 1 well-differentiated (G1) EC cells. In addition, HEC-1A and KLE are both type 2 ECs, which are originally established from moderately differentiated (G2) and poorly differentiated (G3) endometrial adenocarcinomas, respectively [5]. On the other hand, we sorted out the following experimental limitations: First, in order to clarify whether hinokitiol can promote apoptosis by promoting ROSs, N-acetylcysteine (a ROS scavenger) should be used to further examine the results of apoptosis. Similarly, U0126 or PD98059 (inhibitors of ERK) should be administered to explore their effects on the expression of apoptosis-related proteins induced by hinokitiol. Finally, we also regret the lack of animal experiments, but due to the limited funds, we could only perform preliminary cancer cell suppression studies and provide a clinical basis for in-depth exploration of hinokitiol in endometrial cancer in the future.

## 4. Materials and Methods

### 4.1. Preparation of Hinokitiol

Hinokitiol (C_10_H_12_O_2_; CAS number: 499-44-5; purity ≥ 99%; Sigma-Aldrich, St. Louis, MO, USA) is a white to yellow powder that is insoluble in water with a molecular weight of 164.2 kDa. The powder was dissolved in dimethyl sulfoxide (DMSO; Sigma-Aldrich, St. Louis, MO, USA) to prepare a stock solution of 100 mM and was stored at −20 °C until use. A vehicle solvent (0.05% DMSO) was added to the control group.

### 4.2. Cell Lines and Culture

The Ishikawa human endometrial adenocarcinoma cell line was obtained from the European Collection of Authenticated Cell Culture (ECACC; Salisbury, UK), and it was cultured in MEM (Caisson Labs, Smithfield, UT, USA). The HEC-1A human endometrial cancer cell line was purchased from the Food Industry Research and Development Institute (FIRDI; Taiwan, ROC) and Culture Collection and Research Center (CCRC; Taiwan, ROC), and it was cultured in McCoy’s 5A (Sigma-Aldrich, St. Louis, MO, USA). The KLE human endometrial cancer cell line was purchased from the American Type Culture Collection (ATCC; Manassas, VA, USA), and it was cultured in DMEM/Ham’s F-12 (Caisson Labs, Smithfield, UT, USA). All cells were maintained in complete growth medium containing 10% fetal bovine serum (FBS; CORNING, Manassas, VA, USA), and they were incubated at 37 °C with 5% CO_2_. The cell culture medium was collected and checked for mycoplasma by using the EZ-PCR-Mycoplasma Test Kit (Biological Industries, Cromwell, CT, USA).

### 4.3. Cell Viability Assays

The influence of hinokitiol on the cell viability of the HEC-1A, Ishikawa, and KLE cell lines was analyzed with an (3-(4,5-Dimethylthiazol-2-yl)-2,5-diphenyltetrazolium bromide) (MTT) assay (Sigma-Aldrich, St. Louis, MO, USA). Briefly, the cells were seeded in 96-well plates (3 × 10^3^ per well) and treated with various dosages (1, 5, 10, 25, and 50 μM) of hinokitiol or DMSO (0.05 %) alone as a vehicle control for 48 h. At the indicated time points, the medium was replaced with 100 μL of fresh medium containing 1% serum with 0.5 mg/mL MTT. Following a three-hour incubation at 37 °C, formazan crystals were solubilized with 100 μL of DMSO. The absorbance levels for each sample at 570 nm with a reference wavelength of 630 nm were measured by using a microplate reader (Bio-Tek, Winooski, VT, USA). The data were duplicated at least three times. The average inhibitory concentration (IC50) of hinokitiol was determined by using GraphPad Prism version 9.0 (GraphPad, San Diego, CA, USA).

### 4.4. Western Blotting Analysis

HEC-1A, Ishikawa, and KLE cells were seeded into 10 cm^2^ dishes and treated with a gradient of concentrations (1, 5, 10, 25, and 50 μM) of hinokitiol or DMSO (0.05%) alone as a vehicle control for 48 h. After being digested with 0.25% trypsin (*w*/*v*), the cells were harvested by centrifugation and washed once with cold phosphate-buffered saline (PBS). Cell lysates were lysed with 100 μL of lysis buffer (mixed with a protease and phosphatase inhibitor cocktail (Roche, Basel, Switzerland)). Samples containing 20 μg of proteins were subjected to sodium dodecyl sulfate polyacrylamide gel electrophoresis (SDS-PAGE) gels and transferred to a polyvinylidene difluoride (PVDF, 0.45 or 0.22 µm, MilliporeSigma, Burlington, MA, USA) membrane (for 100 min at 100 V). The membranes were blocked with 5% bovine serum albumin (BSA, BioShop, Burlington, ON, Canada) for 1 h and were then probed with primary antibodies at 4 °C overnight. Membranes were incubated with the corresponding goat anti-rabbit/mouse antibody IgG (1:10000, Abcam, Cambridge, UK) for 1 h. After reacting with electrochemiluminescence (ECL; Thermo Fisher Scientific) in the dark, chemiluminescence imaging was performed with an eBlot Touch Imager^TM^ (eBlot Photoelectric Technology, Shanghai, China). Densitometric estimations were quantified with the Image J software (National Institutes of Health, NIH, Bethesda, MD, USA). Each experiment was carried out in triplicate, and the results are expressed as means ± SD for each treatment group. All of the raw data from the use of Western blotting are provided in Figure S1 (Supplementary Materials). The primary antibodies, including anti-PARP, anti-Caspase-3, anti-Bax, anti-LC3B, anti-p62/SQSTM1, anti-p-P53, anti-pERK1/2, and anti-ERK1/2, were purchased from Cell Signal Technology (Beverley, MA, USA); Bcl-2 was purchased from Santa Cruz Biotechnology (Dallas, TX, USA); CDK4 and cyclin D1 were purchased from Abcam (Cambridge, UK); anti-horseradish peroxidase (HRP)-conjugated glyceraldehyde 3-phosphate dehydrogenase (GAPDH) was purchased from Proteintech (Rosemont, IL, USA). The dilution concentrations of all antibodies used in the Western blotting are provided in Table S1.

### 4.5. Flow Cytometry Analysis

An FITC Annexin V Apoptosis Detection Kit I was purchased from BD Biosciences (Franklin Lakes, NJ, USA). HEC-1A, Ishikawa, and KLE cells were seeded into 6-well plates and treated with a gradient of concentrations (1, 5, 10, 25, and 50 μM) of hinokitiol or DMSO (0.05%) alone as a vehicle control for 48 h. After being digested with 0.25% (*w*/*v*) trypsin, all of the cells were harvested by centrifugation and washed twice with cold PBS. The pellets were resuspended in 100 μL of 1× binding buffer and incubated with 2 μL of Annexin V-FITC and 2 μL of PI for 15 min at room temperature in the dark according to the manufacturer’s instructions. After the reaction was completed, the 1× binding buffer was replenished to 500 μL in each sample tube. Cell apoptosis was detected within 1 h by using an Attune™ NxT Flow Cytometer (Thermo Fisher Scientific, Waltham, MA, USA). Each experiment was carried out in triplicate, and the results are expressed as means ± SD for each treatment group.

### 4.6. Measurement of Intracellular Reactive Oxygen Species (ROSs)

The general oxidative stress indicator 6-carboxy-2′,7′-dichlorodihydrofluorescein diacetate (carboxy-H_2_DCFDA) was purchased from Invitrogen™ (Thermo Fisher Scientific, Waltham, MA, USA). To quantitatively assess intracellular ROSs, the cells were treated with different concentrations (1, 5, 10, 25, and 50 μM) of hinokitiol or DMSO (0.05 %) alone as a vehicle control in 6-well plates for 48 h. After being digested with 0.25% (*w*/*v*) trypsin, the cells were harvested by centrifugation, washed twice with PBS, and then incubated with 20 µM carboxy-H_2_DCFDA (6-carboxy-2′,7′-dichlorodihydrofluorescein diacetate) in culture medium without serum at 37 °C in the dark for 45 min. After the reaction was completed, the stained cells were washed once with PBS to remove the residual dye. Except for the positive control group (H_2_O_2_), for ROSs to be effectively induced, the rest of the samples were kept on ice for later use. For the H_2_O_2_ positive control treatment, the medium was refreshed with reduced serum (1%) containing 1 mM H_2_O_2_ and incubated for 30 min. ROS levels were assessed with an Attune™ NxT Flow Cytometer (Thermo Fisher Scientific, Waltham, MA, USA) using the FL1 channel with an Ex/Em of ~492–495/517–527 nm. Each experiment was carried out in triplicate, and the results are expressed as means ± SD for each treatment group.

### 4.7. Statistical Analysis

The data were analyzed by using SigmaPlot version 12.5 (SoftHome, Taipei, Taiwan, China) or GraphPad Prism version 9.0 (GraphPad, San Diego, CA, USA), and they are expressed as the mean ± standard deviation (SD). Statistical significance was evaluated by using two-tailed Student’s *t*-tests (two groups). A difference between two means was considered statistically significant when *p* < 0.05 or highly significant when *p* < 0.001.

## 5. Conclusions

**Figure 8:**
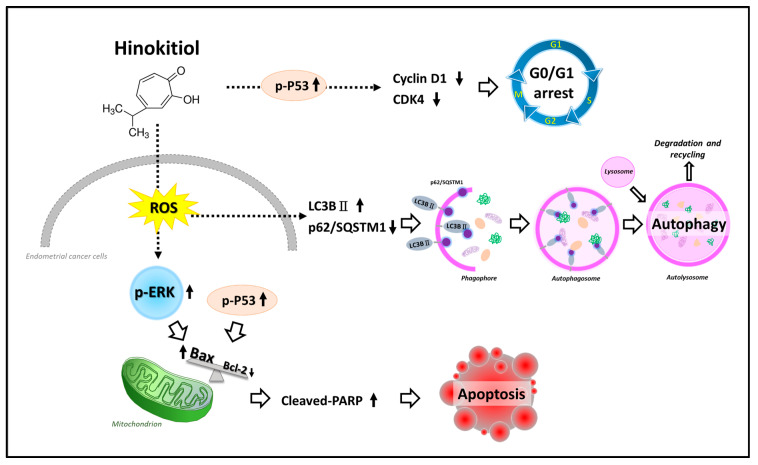
A schematic representation of the hypothetical mechanisms of the role of hinokitiol in suppressing endometrial cancer. The upward arrow represents “enhancement”; the downward arrow represents “inhibition”; the dashed arrow represents “the possible pathway induced by hinokitiol”; The unfilled arrow represents the “direction of the mechanism”.

In this study, we investigated the antitumor properties of hinokitiol with respect to human endometrial cancer cells and found that hinokitiol regulates the protein expression of p53 and CDK4/cyclin D1, increases the ROS level, and activates apoptosis and autophagy through the ERK1/2 pathway in Ishikawa, HEC-1A, and KLE cells (Figure 8). Overall, this study might provide fundamental knowledge for understanding the antitumor activity of hinokitiol in endometrial cancer cells, and it is hoped that hinokitiol can potentially be used as an alternative drug for endometrial cancer in the future, pending further studies.

## Data Availability

Data can be available upon request.

## Supplementary Materials

The following are available online at https://www.mdpi.com/article/10.3390/ijms22158268/s1, Table S1: The dilution concentrations of all antibodies used in Western blotting. Figure S1: The raw data from Western blotting.

## References

[1] Brooks R.A., Fleming G.F., Lastra R.R., Lee N.K., Moroney J.W., Son C.H., Tatebe K., Veneris J.L. (2019). Current recommendations and recent progress in endometrial cancer. CA Cancer J. Clin..

[2] Lai J.C.-Y., Weng C.-S., Huang S.-M., Huang N., Chou Y.-J., Wang C.-C., Wang K.-L. (2017). Incidence and lifetime risk of uterine corpus cancer in Taiwanese women from 1991 to 2010. Taiwan. J. Obstet. Gynecol..

[3] Sung H., Ferlay J., Siegel R.L., Laversanne M., Soerjomataram I., Jemal A., Bray F. (2021). Global Cancer Statistics 2020: GLOBOCAN Estimates of Incidence and Mortality Worldwide for 36 Cancers in 185 Countries. CA Cancer J. Clin..

[4] Urick M.E., Bell D.W. (2019). Clinical actionability of molecular targets in endometrial cancer. Nat. Rev. Cancer.

[5] Skok K., Maver U., Gradišnik L., Kozar N., Takač I., Arko D. (2020). Endometrial cancer and its cell lines. Mol. Biol. Rep..

[6] Abdulfatah E., Wakeling E., Sakr S., Al-Obaidy K., Bandyopadhyay S., Morris R., Feldman G., Ali-Fehmi R. (2019). Molecular classification of endometrial carcinoma applied to endometrial biopsy specimens: Towards early personalized patient management. Gynecol. Oncol..

[7] Muller P.A., Vousden K.H. (2014). Mutant p53 in cancer: New functions and therapeutic opportunities. Cancer Cell.

[8] Chen H.Y., Chiang Y.F., Huang J.S., Huang T.C., Shih Y.H., Wang K.L., Ali M., Hong Y.H., Shieh T.M., Hsia S.M. (2021). Isoliquiritigenin Reverses Epithelial-Mesenchymal Transition Through Modulation of the TGF-β/Smad Signaling Pathway in Endometrial Cancer. Cancers.

[9] Haque A., Brazeau D., Amin A.R. (2021). Perspectives on natural compounds in chemoprevention and treatment of cancer: An update with new promising compounds. Eur. J. Cancer.

[10] Jayakumar T., Hsu W.H., Yen T.L., Luo J.Y., Kuo Y.C., Fong T.H., Sheu J.R. (2013). Hinokitiol, a natural tropolone derivative, offers neuroprotection from thromboembolic stroke in vivo. Evid. Based Complementary Altern. Med..

[11] Domon H., Hiyoshi T., Maekawa T., Yonezawa D., Tamura H., Kawabata S., Yanagihara K., Kimura O., Kunitomo E., Terao Y. (2019). Antibacterial activity of hinokitiol against both antibiotic-resistant and -susceptible pathogenic bacteria that predominate in the oral cavity and upper airways. Microbiol. Immunol..

[12] Lee J.H., Moon J.H., Lee Y.J., Park S.Y. (2017). SIRT1, a Class III Histone Deacetylase, Regulates LPS-Induced Inflammation in Human Keratinocytes and Mediates the Anti-Inflammatory Effects of Hinokitiol. J. Investig. Dermatol..

[13] Wu Y.J., Hsu W.J., Wu L.H., Liou H.P., Pangilinan C.R., Tyan Y.C., Lee C.H. (2020). Hinokitiol reduces tumor metastasis by inhibiting heparanase via extracellular signal-regulated kinase and protein kinase B pathway. Int. J. Med. Sci..

[14] Jayakumar T., Liu C.H., Wu G.Y., Lee T.Y., Manubolu M., Hsieh C.Y., Yang C.H., Sheu J.R. (2018). Hinokitiol Inhibits Migration of A549 Lung Cancer Cells via Suppression of MMPs and Induction of Antioxidant Enzymes and Apoptosis. Int. J. Mol. Sci..

[15] Wang C.C., Chen B.K., Chen P.H., Chen L.C. (2020). Hinokitiol induces cell death and inhibits epidermal growth factor-induced cell migration and signaling pathways in human cervical adenocarcinoma. Taiwan. J. Obstet. Gynecol..

[16] Liu S., Yamauchi H. (2006). Hinokitiol, a metal chelator derived from natural plants, suppresses cell growth and disrupts androgen receptor signaling in prostate carcinoma cell lines. Biochem. Biophys. Res. Commun..

[17] Wei K.-C., Chen R.-F., Chen Y.-F., Lin C.-H. (2019). Hinokitiol suppresses growth of B16 melanoma by activating ERK/MKP3/proteosome pathway to downregulate survivin expression. Toxicol. Appl. Pharmacol..

[18] Shih Y.H., Chang K.W., Hsia S.M., Yu C.C., Fuh L.J., Chi T.Y., Shieh T.M. (2013). In vitro antimicrobial and anticancer potential of hinokitiol against oral pathogens and oral cancer cell lines. Microbiol. Res..

[19] Elmore S. (2007). Apoptosis: A review of programmed cell death. Toxicol. Pathol..

[20] Dikic I., Elazar Z. (2018). Mechanism and medical implications of mammalian autophagy. Nat. Rev. Mol. Cell Biol..

[21] Tanaka S., Tak Y.S., Araki H. (2007). The role of CDK in the initiation step of DNA replication in eukaryotes. Cell Div..

[22] Suryadinata R., Sadowski M., Sarcevic B. (2010). Control of cell cycle progression by phosphorylation of cyclin-dependent kinase (CDK) substrates. Biosci. Rep..

[23] Chen J., Ko J., Kim J.T., Cho J.S., Qiu S., Kim G.D., Auh J.H., Lee H.J. (2019). β-Thujaplicin inhibits basal-like mammary tumor growth by regulating glycogen synthase kinase-3β/β-catenin signaling. Food Funct..

[24] Hernández Borrero L.J., El-Deiry W.S. (2021). Tumor suppressor p53: Biology, signaling pathways, and therapeutic targeting. Biochim. Biophys. Acta Rev. Cancer.

[25] Strasser A., O’Connor L., Dixit V.M. (2000). Apoptosis signaling. Annu. Rev. Biochem..

[26] Zhang G., He J., Ye X., Zhu J., Hu X., Shen M., Ma Y., Mao Z., Song H., Chen F. (2019). β-Thujaplicin induces autophagic cell death, apoptosis, and cell cycle arrest through ROS-mediated Akt and p38/ERK MAPK signaling in human hepatocellular carcinoma. Cell Death Dis..

[27] Ciuffa R., Lamark T., Tarafder A.K., Guesdon A., Rybina S., Hagen W.J., Johansen T., Sachse C. (2015). The selective autophagy receptor p62 forms a flexible filamentous helical scaffold. Cell Rep..

[28] Srinivas U.S., Tan B.W.Q., Vellayappan B.A., Jeyasekharan A.D. (2019). ROS and the DNA damage response in cancer. Redox Biol..

[29] Xu Y., Wang S., Miao Q., Jin K., Lou L., Ye X., Xi Y., Ye J. (2017). Protective Role of Hinokitiol Against H_2_O_2_-Induced Injury in Human Corneal Epithelium. Curr. Eye Res..

[30] Zhang F., Zhang Y.Y., Sun Y.S., Ma R.H., Thakur K., Zhang J.G., Wei Z.J. (2020). Asparanin A from Asparagus officinalis L. Induces G0/G1 Cell Cycle Arrest and Apoptosis in Human Endometrial Carcinoma Ishikawa Cells via Mitochondrial and PI3K/AKT Signaling Pathways. J. Agric. Food Chem..

[31] Li X.L., Ma R.H., Ni Z.J., Thakur K., Cespedes-Acuña C.L., Wang S., Zhang J.G., Wei Z.J. (2021). Dioscin inhibits human endometrial carcinoma proliferation via G0/G1 cell cycle arrest and mitochondrial-dependent signaling pathway. Food Chem. Toxicol..

[32] Wu G.S. (2004). The functional interactions between the p53 and MAPK signaling pathways. Cancer Biol. Ther..

