# Supplementary material for: Hinokitiol Exhibits Antitumor Properties through Induction of ROS-Mediated Apoptosis and p53-Driven Cell-Cycle Arrest in Endometrial Cancer Cell Lines (Ishikawa, HEC-1A, KLE)
Source: Int J Mol Sci. 2021 Jul 31;22(15):8268. doi: 10.3390/ijms22158268 (PMC8348875; doi:10.3390/ijms22158268)

Supplemant data-Table. 1. The dilution concentrations of all antibodies used in western blotting

Item	Number	Brand	Dilution
Phospho-p53 (Ser15)	#9284	Cell signaling	1000
CDK4	#12790	Cell signaling	1000
Cylcin D1	ab134175	Abcam	500
PARP	#9532	Cell signaling	1000
BAX	#2772	Cell signaling	1000
BCL-2	sc-7382	SANTA CRUZ	500
Caspase-3	#9662	Cell signaling	1000
LC3B	#3868	Cell signaling	1000
SQSTM1/p62	#39749	Cell signaling	1000
p44/42 MAPK (Erk1/2)	9102s	Cell signaling	1000
Phospho-p44/42 MAPK (Erk1/2) (Thr202/Tyr204)	9101s	Cell signaling	1000
Goat Anti-Rabbit IgG H&L (HRP) preadsorbed	ab7090	Abcam	10000
Goat Anti-Mouse IgG H&L (HRP) preadsorbed	ab97040	Abcam	10000
GAPDH	HRP-60004	Proteintech	10000

Supplemant data- Figure. 1. The raw data using western blotting.

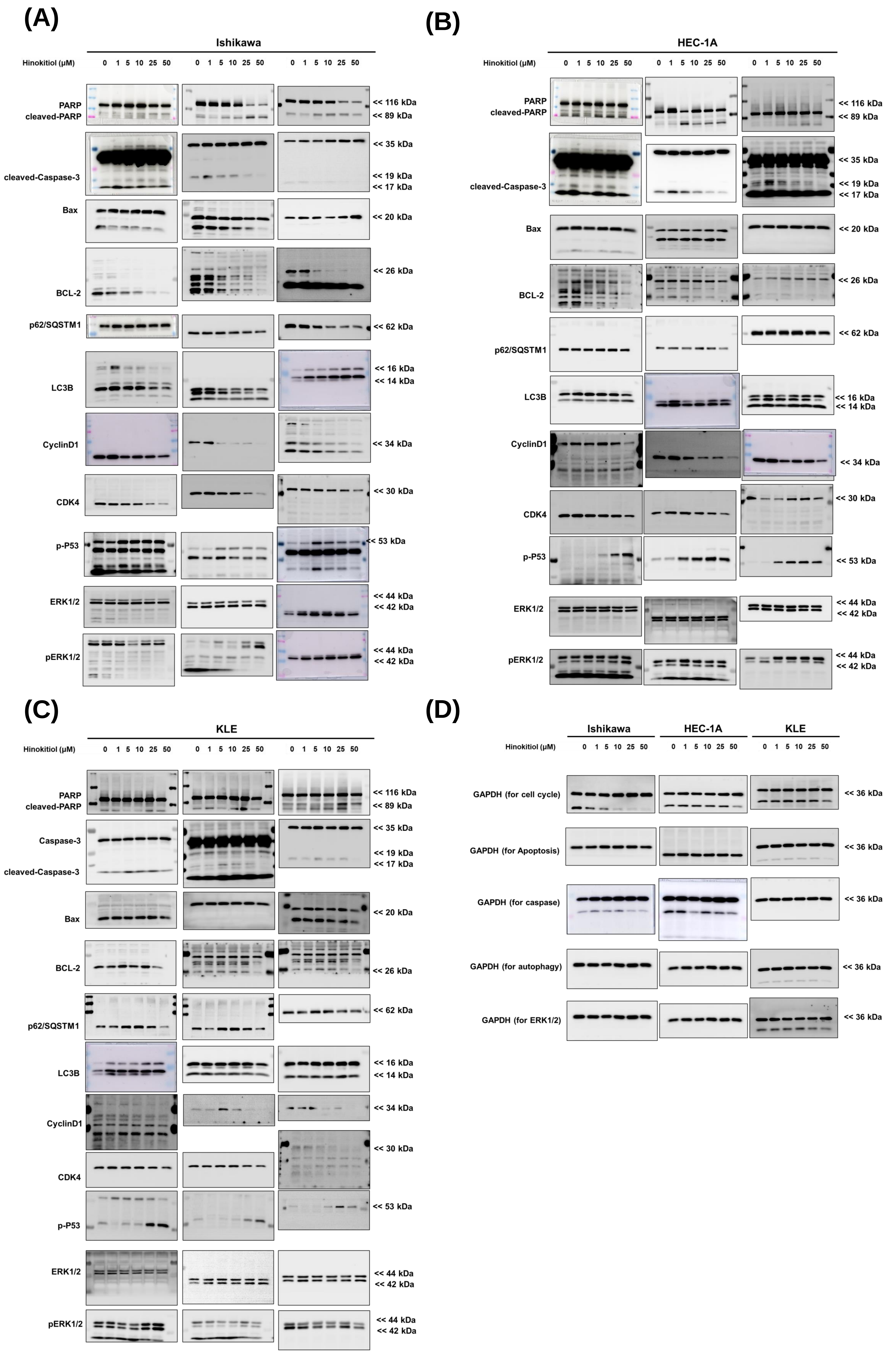

Supplement: Supplementary file 1 [file ijms-22-08268-s001.zip › ijms-1287251-supplementary.pdf]
